# Public health surveillance in countries hosting displaced people from Ukraine

**DOI:** 10.2807/1560-7917.ES.2022.27.22.2200430

**Published:** 2022-06-02

**Authors:** Julien Beauté, Piotr Kramarz

**Affiliations:** 1 European Centre for Disease Prevention and Control (ECDC), Stockholm, Sweden

**Keywords:** public health surveillance, migrants, armed conflicts, Europe

More than 6 million people have fled Ukraine in reaction to the Russian invasion starting on 24 February 2022, most of them to countries in the European Union/European Economic Area (EU/EEA) [1]. Of the 3.5 million who arrived in Poland, it is estimated that approximately 2 million could remain in the country by December 2022. Other countries, such as Czechia, Germany and Romania, have also received hundreds of thousands of displaced people fleeing Ukraine. This large number of people poses challenges for public health surveillance, which generally aims to quantify the magnitude of health problems and monitor the impact of interventions [2].

One important basic assumption underlying surveillance is that the population under surveillance remains relatively stable over time. This is clearly not the case in the current crisis where large groups of displaced people move from one area and country to another and sometimes back in a short period of time. These people are evidently exposed to risks due to conditions faced during displacement and there is a risk of discontinuity of care and possible increase of severe disease outcome [3]. Poor temporary living conditions, combined with potentially low vaccination coverage, may constitute a risk for outbreaks of vaccine-preventable diseases [4]. Taken together, this means that the current crisis may impact surveillance data and indicators and it is essential to address this to maintain high quality data and fulfil surveillance objectives.

The health burden before the Russian invasion in Ukraine was higher compared with EU/EEA countries for both communicable diseases such as HIV and tuberculosis and for non-communicable diseases—for example, the estimated age-adjusted death rate for ischaemic heart disease was six times higher than in EU countries [5]. A good knowledge of the disease burden in displaced populations is important to understand the epidemiological situation and surveillance data are valuable when linked to services as they help inform resource allocation. This will be of particular importance since large numbers of displaced people may put additional pressure on local healthcare systems, as reported in Poland [6]. Other relevant surveillance objectives include outbreak detection and evaluation of interventions such as vaccination campaigns.

Since routine surveillance systems rely on data collected by healthcare providers, countries need to make sure all those displaced have access to healthcare, including laboratory testing and treatment [7]. It is further relevant that professionals who provide healthcare to displaced people can also report to the existing surveillance system, especially if healthcare is not provided by pre-existing community services, as is the case for non-governmental organisations (NGO) operating in reception centres. At border crossing points and reception there is a need to raise awareness among medical teams about the importance of reporting notifiable diseases/conditions and how to do so, while keeping in mind that some international NGO healthcare staff may not speak the language of the country in which they work. However, routine surveillance systems are expected to play a major role since most displaced people in Poland and other receiving countries are living in the general community.

When collecting surveillance data, it is imperative to collect information on migration status to be able to identify cases among displaced people and be able to identify specific risks where public heath action can be taken. Ideally, this information should be captured following agreed definitions, such as those listed in the *Glossary on migration* published by the International Organisation for Migration [8], which distinguishes migrants, refugees, displaced people and asylum seekers. Unfortunately, this information is not routinely captured, and these definitions may not even be fully understood at point of data entry. A report assessing the burden of key infectious diseases affecting migrant populations in the EU/EEA suggested that the best available variable in The European Surveillance System (TESSy) to identify cases in migrants was ‘country of birth’ [9]. In the context of the Ukraine crisis, such a variable would not be able to distinguish Ukrainian citizens living in Poland before the war (estimated at up to 2 million), or other Ukraine-neighbouring countries, from those who fled after the start of the Russian invasion unless the ‘date of entry’ were also reported.

If displaced persons live in large congregate facilities such as reception centres, it may be useful to collect additional information on the setting of infection. If these data are reported timely, they can be used to trigger an early warning and prompt control measures as well as help determine which settings are associated with a higher risk of infection. In such centres, syndromic surveillance systems could complement routine surveillance to rapidly detect signals or public health threats [10].

Calculation of disease rates is crucial to make valid comparisons between countries and across different groups or over time. Since most displaced persons from Ukraine are living in the general community, they are in fact part of the population under surveillance. Yet, their large numbers will not immediately be reflected in population denominator data. This may distort rates, especially in females and younger age groups which are overrepresented among the displaced populations from Ukraine. If denominator data by country of origin are available, it may be of interest to calculate rates by origin. For example, an analysis of tuberculosis surveillance data in the EU/EEA showed that the decrease in the notification rate over time was higher in native residents than in those of foreign origin [11]. It may be challenging to obtain sound population denominator data if the displaced population fluctuates greatly. For any populations staying for short periods, the challenges are similar to those encountered when dealing with travel-associated infections, as illustrated for coronavirus disease (COVID-19) surveillance [12]. When displaced people return to Ukraine in the coming months or years, it may be useful to be able to exclude respective cases from long time-series analyses.

Migrants may be subject to specific programmes that may impact surveillance data, such as latent tuberculosis infection screening [13]. This should be considered when interpreting surveillance data. For diseases with a long duration such as HIV or tuberculosis, it may be important to determine whether the diagnosis was known before the notification and therefore consider variables collecting this information.

If there are frequent and possibly automated analyses of surveillance data, signals of outbreaks in specific settings could trigger early alert and response mechanisms. If case numbers among displaced populations are small, it is probably unnecessary to stratify analyses by country of origin. Conversely, if numbers of people are large and especially if their characteristics differ from the rest of the population, it may be advisable to carry out subset analyses as this could help understand if displaced persons are exposed to increased risk of some infections. When considering such analyses, stigmatisation of refugees should be avoided. Analyses of data related to the current crisis should help inform public health interventions. Previous analyses of EU/EEA surveillance data suggested, for example, that the proportions of migrants who acquired HIV in the destination country depended on their country of origin [14]. Other indicators, such as route of transmission or time from migration to diagnosis, also depended on region of origin. Such findings are valuable for both monitoring the epidemic and designing testing and prevention strategies.

In conclusion, good public health surveillance is essential and the large number of displaced people from Ukraine, following the Russian invasion, poses several challenges to the surveillance systems in EU/EEA countries. Integration of this population in the general community and routine surveillance systems able to monitor diseases by migration status is key to effective disease prevention and control. From a surveillance perspective, collecting information on migration status will be increasingly important to detect any specific risks to which displaced populations from Ukraine settling in the EU/EEA may be exposed. The combination of country of origin and date of entry is probably the best option for data analysis and would avoid possible stigmatisation associated with the wording ‘migrant’ or ‘refugee’ while allowing specific public health action.
